# Molecular diagnosis of glycogen storage disease type I: a review

**Published:** 2019-01-30

**Authors:** Zahra Beyzaei, Bita Geramizadeh

**Affiliations:** 1 Shiraz Transplant Research Center (STRC), Shiraz, Iran; 2 Department of Pathology, Shiraz University of Medical Sciences, Shiraz, Iran

**Keywords:** DNA mutational analysis, glycogen storage disease type I, glucose-6-phosphatase-alpha, glucose-6-phosphate translocase, molecular genetics diagnosis

## Abstract

Glycogen storage disease type I (GSD I) is a relatively rare metabolic disease with variable clinical intensity. It is caused by deficient activity of the glucose 6-phosphatase enzyme (GSD Ia) or a deficiency in the microsomal transport proteins for glucose 6-phosphate (GSD Ib). We searched the most recent English literature (1997-2017) regarding any article with the key word of “glycogen storage disease type I” in PubMed, Science Direct, Scopus, EMBASE, and Google Scholar. We will present all of the published articles about the molecular genetic characteristics and old-to-new diagnostic methods used to identify GSD I in regard of methodology, advantages and disadvantages. Diagnosis of GSD type I and its variants is challenging because it is a genetically heterogeneous disorder. Many molecular methods have been used to diagnose GSD I most of which have been based on mutation detection. Therefore, we discuss complete aspects of all of the molecular diagnostic tests, which have been used in GSD type I so far. With the advent of high throughput advanced molecular tests, molecular diagnosis is going to be an important platform for the diagnosis of storage and metabolic diseases such as GSD type I. Next-generation sequencing, in combination with the biochemical tests and clinical signs and symptoms create an accurate, reliable and feasible method. It can overcome the difficulties by the diagnosis of diseases with broad clinical and genetic heterogeneity.

## Introduction to History

Glycogen storage diseases (GSDs) are a group of metabolic disorders determined by the accumulation of glycogen in different tissues. GSDs are caused by the enzyme deficiencies effect on glycogen synthesis, glycogen breakdown or glycolysis (glucose breakdown), typically within muscles and/or liver cells. The disorders were numbered as they were discovered which classified chronologically by GSD type I (von Gierke disease) to GSD type XI (Chen, 2003[4]). Majority of glycogen storage diseases are autosomal recessive. The cumulative incidence of these diseases is approximately 1 in every 20,000 live births (Dambska et al., 2017[9]).

GSD (Glycogen storage disease) type I or von Gierke disease is the second most common type of GSDs. GSD type I typically presents in early childhood. Von Gierke disease is an autosomal recessive disorder. There is no report regarding ethnic difference in the incidence of GSD type I, however there are different types of mutations in Caucasian, Hispanic, Asian and Jewish populations (Ekstein et al., 2004[10]). The overall incidence of this disease is about 1 to 100,000, although its prevalence in the Ashkenazi Jewish population is relatively high (prevalence 1/20,000) (Ekstein et al., 2004[10]). This disease is more prevalent in the populations with high consanguineous marriages (Bagheri Lankarani et al., 2013[1]; Geramizadeh and Malek-Hosseini, 2017[15]). 

Historically, in 1929, Edgar von Gierke was the first to describe a glycogen storage disease (GSD), which initially was named in his honor (Schall, 1932[49]; Unselm, 1932[55]; Matern et al., 2002[37]). Later, after 20 years, Cori and Cori (1952[8]) found glucose-6-phosphatase (G6Pase; EC 3.1.3.9) to be deficient in patients with von Gierke disease. Five subtypes of GSD I (Ia, IaSP, Ib, Ic and Id) have been classified so far (Veiga-da-Cunha et al., 2000[58]). However, nowadays most studies have divided this disease into two general categories: GSD Ia and GSD I non-a. Generally, GSD I is an important type of GSDs regarding the need for liver transplantation to overcome the enzyme deficiency (Senior and Loridan, 1968[50]).

The purpose of this narrative review is to describe recent developments in GSD I in regard of molecular diagnostic tools for the detection of mutations. For this purpose, we searched the most recent English literature (1997-2017) regarding any article with the key word of “glycogen storage disease type I” in PubMed, Science Direct, Scopus, EMBASE, and Google Scholar.

## Clinical Manifestation

GSD I is characterized by accumulation of glycogen and fat in the liver and kidneys, leading to hepatomegaly and renomegaly. The clinical manifestations of von Gierke disease include growth retardation, hepatomegaly, hypoglycemia, lactic acidemia, hyperuricemia, osteoporosis and hyperlipidemia (Cori and Cori, 1952[8]). The patients represent typical facial features (round, full-cheeked face) and frequently show ovarian cysts, liver adenomas with a tendency to malignant transformation and enlarged kidneys. Approximately 80 % of patient with GSD I are categorized as type Ia and 20 % as type Ib (Lei et al., 1993[32]).

The clinical presentations of GSD Ib is quite similar to that of GSD Ia, however the symptoms of the patients with GSD Ia, such as hepatomegaly, a characteristic ''doll-like'' face, short stature, and chronic fatigue are more severe. Unlike patients with GSD-Ia, most patients with GSD-Ib also suffer from neutrophil dysfunction and neutropenia, lead to frequent bacterial infections. Clinical differential diagnosis of GSD-Ia from Ib is difficult because neutropenia is sometimes periodic or never develops in GSD-Ib (Cori and Cori, 1952[8]; Lei et al., 1993[32]).

## Molecular Pathogenesis

In terms of molecular genetics, the gene of G6Pase identified by Lei and co-workers (1993[32]), which spans 12.5 kb on chromosome 17q21 consists of 5 exons. The catalytic subunit of microsomal G6Pase plays a crucial role in glycogenolysis and gluconeogenesis catalyzing the last step of both metabolic pathways. For the first time in 1997, a gene (*G6PT1*) was codified the enzyme translocase and mutations within this gene in GSD Ib patients were described (Gerin et al., 1997[16]). *G6PT1* maps to chromosome 11q23 and consists of 9 exons (Matern et al., 2002[37]). It has been suggested that the enzyme uses one transport system (*G6PT1*) to translocate glucose-6-phosphate (G6P) from the cytosol to the lumen of the endoplasmic reticulum (ER) and two other transport systems to transport the reaction products phosphate (Pi) and glucose (*G6PT2* and *G6PT3* respectively) to the cytosol (Matern et al., 2002[37]). 

In 1994, other subtypes have also been introduced and labeled as GSD IaSP, Ic, and Id (Matern et al., 2002[37]). But with advances in molecular genetic methods, the patients with GSD IaSP have discovered to have mutations in *G6PC* and most patients with GSD Ic and Id have mutations in the *G6PT1* gene *SLC37A4*, therefore GSD I is now divided into two general subtypes GSD Ia and GSD I non-a, respectively (Lei et al., 1995[31]; Veiga-Da-Cunha et al., 1999[57]). However the GSD I non-a is divided to GSD Ib (MIM 232220) and 1c (MIM 232240) in which the transport of G6P or PPi/Pi is impaired. GSD 1c is distinguished from GSD 1b by a decrease in enzyme latency in isolated microsomes with enhancing G6P concentrations (Veiga-Da-Cunha et al., 1999[57]; Kishnani et al., 2014[25]; Janecke et al., 2001[22]). Among these subtypes, two of them are more important; GSD-Ia, due to a deficiency in the catalytic subunit of glucose-6-phosphatase-alpha (G6Pase-α or *G6PC*) and GSD-Ib, a deficiency in the glucose-6-phosphate (G6P) transporter (*G6PT*) (Janecke et al., 2001[22]).

## Clinicopathologic Diagnosis

The final and conclusive diagnosis of GSD needed pathologic diagnosis in combination with biochemical and clinical findings; however, liver biopsy is an invasive procedure (Kishnani et al., 2014[25]). Practically tentative diagnosis of GSD I can be made based on the clinical characteristic and biochemical findings. In the new molecular diagnostic era, researchers are trying to find molecular genetic methods for accurate and quick diagnosis of the disease. Different molecular diagnostic methods have been used so far, some of which are challenging and have cons and pros. However, gene mutational analysis allows non-invasive and accurate way of diagnosing type Ia and Ib patients (Janecke et al., 2001[22]).

## Molecular Genetic Diagnostic Methods

Prompt and accurate diagnosis is the most important point for the proper treatment of metabolic diseases. Diagnosis of GSD I is sometimes complicated because there are common features between GSD I and III, including hepatomegaly, hypoglycemia, and hyperlipidemia. Validated and clinically useful tools with a positive predictive value > 90 % are necessary for the diagnosis of GSD I. The diagnosis of GSD I currently relies on clinical features, pathologic diagnosis of liver biopsy, biochemical, and molecular genetic tests (Chen, 2003[4]; Geramizadeh and Malek-Hosseini, 2017[15]; Matern et al., 2002[37]). 

The gold standard for the diagnosis of GSD type I consists of evaluation of liver enzyme deficiency (glucose 6-phosphatase catalytic activity) and pathologic findings of the liver biopsy in favor of GSD (Bagheri Lankarani et al., 2013[1]; Geramizadeh and Malek-Hosseini, 2017[15]).

One of the best diagnostic methods to identify GSD I is detection of the mutations. Although GSD I is not restricted to any ethnic population, mutations unique to a specific race were identified (Chou et al. 2017[7]). So mutations in the *G6PC* or *G6PT1* gene unique to Caucasian, Hispanic, Chinese/Japanese/ Korean, and Jewish GSD I patients have been described, suggesting separate ethnic founder effects for some mutations (Table 1(Tab. 1)). For this reason, various diagnostic methods have been used for mutation detection. Molecular methods for identification of the disease-causing mutations could be classified as methods for known and unknown mutations (Nejat and Rabbani, 2013[40]). Later, this review focuses on all the published and reported molecular methods identified and used for the diagnosis of this disorder, as summarized in Table 2(Tab. 2) (References in Table 2: Barkaoui et al., 2007[2]; Chiang et al., 2000[5]; Choi et al., 2017[6]; Ekstein et al., 2004[10]; Eminoglu et al., 2013[11]; Ezgu et al., 2014[12]; Fujii et al., 2000[13]; Galli et al., 1999[14]; Gu et al., 2014[17]; Hou et al., 1999[19]; Ihara et al., 1998[20]; Janecke et al., 1999[22]; Ki et al., 2004[24]; Kojima et al., 2004[26]; Kozák et al., 2000[28]; Lam et al., 1998[29]; Lam et al., 2000[30]; Liang et al., 2013[33]; Lu et al., 2016[34]; Mahmoud et al., 2017[35]; Marcolongo et al., 1998[36]; Miltenberger-Miltenyi et al., 2005[38]; Nakamura et al., 2001[39]; Okubo et al., 1997[42]; Qiu et al., 2013[44]; Qiu et al., 2011[43]; Rake et al., 2000[46]; Reis et al., 2001[47]; Santer et al., 2000[48]; Seydewitz and Matern, 1999[51]; Tamhankar et al., 2012[53]; Trioche et al., 1999[54]; Veiga-da-Cunha et al., 1999[57]; Wong et al., 2001[60]; Xu et al., 2010[61]; Yuen et al., 2002[62]; Zheng et al., 2015[63]; Zhu et al., 2012[64]).

### Analysis based on unknown mutations

#### Single-strand conformation polymorphism (SSCP) and Heteroduplex analysis (HD)

SSCP and HD methods are used together to increase the accuracy. The principle of both these electrophoretic methods is based on the fact that single-stranded DNA has a specific conformation (Veiga-Da-Cunha et al., 1999[57]). Transformed conformation due to a single base alteration in the sequence can cause single-stranded DNA to migrate differently under electrophoresis conditions (Marcolongo et al., 1998[36]; Galli et al., 1999[14]). After the report of the first mutations in the glucose 6-phosphatase gene as the cause of GSD type I, in the 90's (Lei et al., 1993[32]; Gerin et al., 1997[16]), multiple molecular diagnostic methods have been introduced. One of the first reports of mutation screening of GSD Ia, Ib was performed by both Single-strand conformation polymorphism (SSCP) and Heteroduplex analysis (HD) to develop a feasible diagnostic method (Veiga-Da-Cunha et al., 1999[57]). It is known that SSCP analysis is one of the simplest and most popular techniques for mutation detection and genotyping so many reports in the late 90s were published. Marcolongo et al. (1998[36]) found mutations in six out of seven GSD 1b patients by SSCP analysis and they suggested SSCP and HD as a useful tool for genetic diagnostic procedure for searching (new) mutations in GSD 1b patients. They concluded that mutations in the promoter or other untranslated regions of the gene cannot be excluded. Veiga-da-Cunha et al. (1999[57]) have performed mutation screening for 23 families of different populations diagnosed as having GSD I non-a by both SSCP and HD analysis to optimize the detection procedure, to detect 16 new mutations (among the 16 new mutations found, seven were substitutions) as well as nine that had been previously described. Ten of the new mutations were detectable by SSCP alone and the remaining six were found by HD analysis. They concluded that there is a great variety of mutations in the *GSD Ib *gene and they can be detected by a combination of SSCP and HD analysis. Report of Galli et al. (1999[14]) has confirmed that GSD 1b and 1c are due to mutations in the same gene, i.e. the *G6PT* gene. They came to this conclusion by performing SSCP and/or DNA sequencing in 14 Italian patients. Seydewitz et al. (1999[51]) analyzed 40 German paitents with GSD Ia by SSCP method. They have performed sequencing in all 5 exon for certainty in any case that none or only one mutation was detected by SSCP analysis. Also Hiraiwa et al. (1999[18]) showed that by SSCP a total of six different mutations. One missense mutation in one allele of the two GSD Ib patients can be detected, but a second *G6PT* mutant allele was only identified after sequencing of five *G6PT* cDNA clones from each patient. Two reports demonstrated that identifying of GSD Ia patients using SSCP prior to automated sequencing of exons can reveal an aberrant SSCP pattern (Rake et al., 2000[46]; Nakamura et al., 2001[39]).

Eventually, even a single base alteration can be detected by the altered mobility of the single-stranded DNA molecule in SSCP. In 2004, in one study the frequency of two prevalent mutations of GSD Ia patient in Caucasian (the Q347X and R83C mutation) was reported to screen the Ashkenazi Jewish population by SSCP method as an accurate and easy technique which leads to a predicted prevalence (1 in 20,000) five times higher than in the general Caucasian population (Ekstein et al., 2004[10]).

According to the above literature, disadvantages of SSCP method include the requirement of highly standardized electrophoretic conditions in order to get constant results. Furthermore, some mutations may remain undetected, and accordingly definite absence of mutation cannot be proven. In HD method single-base substitutions are less stable and excessively sensitive to environmental changes. This fact reduces the sensitivity of this method for this type of mutations, which is frequently found in GSD I (Børresen, 2002[3]; Konstantinos et al., 2008[27]).

#### Denaturing gradient gel electrophoresis (DGGE)

Denaturing gradient gel electrophoresis (DGGE) has been applied for screening of unknown point mutations. Identification of mutations in the *G6PC* gene in Czech and Slovak patients with GSD Ia was reported based on DGGE and PCR/Restriction Enzyme Digestion Analysis method. They detected a total of 9 different mutations, including 6 missense mutations (K76N, W77R, R83C, V166A, G188R, R295C), two deletions (540del5 and 158delC) and one nonsense mutation (Q347X). Three of them have not been described previously and the R83C was the most common mutation among the Czech and Slovak patients (Kozák et al., 2000[28]). This technique is based on differences in the melting behavior of small DNA fragments (200-700 bp) with the usage of specific chemical denaturants (formamide or urea); even a single base substitution can cause such a difference. It is noted that the concentration of denaturants is a crucial and incorrect estimation can cause some mutations to be missed. This method is also time consuming, and biases from DNA extraction and amplification have been reported (Konstantinos et al., 2008[27]).

#### Direct sequencing and next-generation of sequencing (NGS)

The methods described above have some limitations and they only detect the presence of a mutation. So, DNA sequencing usually is necessary to be performed at the same time to determine the nature of the mutation that caused an electrophoretic mobility shift (SSCP or HD) in a given sample on both strands (Veiga-Da-Cunha et al., 1999[57]). Moreover, not all types of point mutations in a specified sequence will cause a detectable change in electrophoretic mobility. Molecular genetic testing via sequencing of the *G6PC *(GSD Ia) and *SLC37A4 *(GSD Ib) full genes can be used for confirming the diagnosis of these diseases precisely (Kishnani et al., 2014[25]; Janecke et al., 2001[22]). Direct sequencing of single-stranded DNA is one of the short, simple and strong methods for mutation detection in genetic disorders such as a GSDs (Lei et al., 1993[32]; Gerin et al., 1997[16]). Direct DNA sequencing was carried out to screen mutations in the coding region, intron/exon junctions and 5' UTRs and 3' UTRs of the *G6PC *and* SLC37A4 *gene (Mahmoud et al., 2017[35]). Since 1997, about 21 studies have been reported mutation identification using the direct sequencing directly or as a complementary diagnostic method especially used in GSD type I (Okubo et al., 1997[42]; Lam et al., 1998[29]; Ihara et al., 1998[20]; Hou et al., 1999[19]; Janecke et al., 1999[21]; Trioche et al., 1999[54]; Chiang et al., 2000[5]; Kozák et al., 2000[28]; Reis et al., 2001[47]; Yuen et al., 2002[62]; Qiu et al., 2013[44]; Ki et al., 2004[24]; Miltenberger-Miltenyi et al., 2005[38]; Qiu et al., 2011[43]; Tamhankar et al., 2012[53]; Liang et al., 2013[33]; Gu et al., 2014[17]; Zheng et al., 2015[63]; Lu et al., 2016[34]; Mahmoud et al., 2017[35], Choi et al., 2017[6]). Among these articles, in 14 reports this method was used to identify GSD Ia directly which concluded that by direct DNA sequencing, novel *G6PC* variations can be identified which expanded the *G6PC* mutation spectrum in the Iranian (Mahmoud et al., 2017[35]), Korean (Ki et al., 2004[24]), Brazilian (Reis et al., 2001[47]), Chinese (Lam et al., 1998[29]; Chiang et al., 2000[5]; Qiu et al., 2013[44]; Gu et al., 2014[17]; Zheng et al., 2015[63]; Lu et al., 2016[34]), Indian (Tamhankar et al., 2012[53]; Karthi et al., 2017[23]), Japanese (Okubo et al., 1997[42]), Czech and Slovak (Kozák et al., 2000[28]), Hungary (Miltenberger-Miltenyi et al., 2005[38]) and French (Trioche et al., 1999[54]) patients, also in 8 studies for GSD I non-a in Hungary (Miltenberger-Miltenyi et al., 2005[38]), Chinese (Yuen et al., 2002[62]; Qiu et al., 2011[43]; Liang et al., 2013[33]) Japanese (Ihara et al., 1998[20]; Hou et al., 1999[19]), Korean (Choi et al., 2017[6]) and Austrian (Janecke et al., 1999[21]) populations. Direct DNA sequencing can usually reveal novel mutations (nonsense, deletion, missense, no-stop,…) and expands knowledge of the *G6PC* and *SLC37A4* mutation spectrum in the populations which provided conclusive genetic evidences for the definitive diagnosis of this disease. Over the past decade newer technologies for DNA sequencing in a massive scale (high throughput) have launched that are referred to as massively parallel or next-generation sequencing (NGS). Direct sequencing has proven extremely successful because of its accuracy and affordability, but it is inappropriate for large-scale screening projects because it has been developed to sequence only one amplified DNA molecule at one time (Nicastro and D'Antiga, 2018[41]). Conversely, NGS has designed to analyze large amounts of sequence data simultaneously, consequently providing different data down to single-base resolution in a rapid, cost-effective and high-throughput fashion on the scale of the whole human genome (Rabbani et al. 2012[45]; Wang et al., 2013[59]). Briefly, NGS involves three basic steps: sample preparation, sequencing, and data analysis (Nicastro and D'Antiga, 2018[41]). Successful sequencing is extremely dependent on the sample preparation procedure, and the type of bases in the regions of interest (ROI). The next step is data analysis including bases calling, read alignment, variant calling and a process of prediction of causality called “annotation” for the genetic variants identification. In recent years, over 10 articles have been published about the application of NGS for the diagnosis of GSDs.

Wang et al. (2013[59]) have developed strategies for using NGS to analyze simultaneous sequencing of the group of candidate genes to facilitate the molecular diagnosis of patients with suspected GSDs. They concluded that NGS can correctly identify all types of mutations and can confirm the molecular diagnosis in patients in whom GSDs were suspected but with the improvement of computational algorithms, bioinformatics analytical tools, sequencing chemistries, interpretation of variants and shortened turnaround time, reliable and fully validated NGS-based clinical tests will eventually become the mainstay of molecular diagnoses (Wang et al., 2013[59]). Vega et al. (2016[56]) have performed genetic analysis of a cohort of 47 Spanish patients and shown the usefulness of NGS in diagnosing GSDs, and in differentiating it from diseases with overlapping phenotypes. Also, Skakic et al. (2018[52]) revealed an unexpectedly high incidence of GSD Ib (1:60 461) with cohort study in Serbian population as the highest frequency in the world. Their patients analyzed by NGS have been successfully genotyped reaching genetic diagnostic rate of 100 %. Their NGS analysis was also shown variants in non-GSD I genes associated with GSD III, VI and IX, as well as with non-GSD associated genes responsible for the cholesteryl-ester storage disease and Shwachman-Diamond syndrome (Skakic et al., 2018[52]). NGS demonstrated 100 % sensitivity and specificity as compared with direct sequencing. The state-of-the-art NGS technology can accurately identify all types of GSD I mutations, which may not be detectable by conventional technology such as direct sequencing. According to published literature, NGS is one of the best techniques in the diagnosis of clinically and genetically heterogeneous disorders such as GSD I, in a cost- and time-efficient manner (Rabbani et al. 2012[45]; Wang et al., 2013[59]; Vega et al., 2016[56]; Skakic et al., 2018[52]).

### Analysis based on known mutation

#### Restriction Fragment Length Polymorphism (RFLP-PCR)

Several approaches have been used for detection of known mutations. These methods usually started with the polymerase chain reaction (PCR) and additional assay steps are performed based on the type of mutation such as RFLP-PCR (Wong et al., 2001[60]). Ihara et al. (1998[20]) reported using PCR-RFLP for detection of two known point mutation (substitutions and transversion mutation) in exon 2 and intron 1 splicing-acceptor site in GSD Ib Japanese patients respectively. Wong et al. (2001[60]) reported the most prevalent mutations in GSD Ia by using RFLP-PCR in Chinese patients of Taiwan which except for R83H, the other mutations have been described only in Asians. Also, Barkaoui et al. (2007[2]) screened two of the most frequent mutations in GSD Ia patients (R83C and R170Q) with a RFLP-PCR technique in Tunisia. Since the majority of Tunisian patients carried R83C and/or R170Q mutations, they proposed direct screening of these mutations with RFLP-PCR technique as an accurate, rapid, valuable and noninvasive tool for diagnosis of GSD Ia in Tunisian (Barkaoui et al., 2007[2]). Therefore, based on this method, it is easy to find the most common and frequent mutations of each population to diagnose the GSD I. It is a simple method for detection of point mutation and single nucleotide polymorphism (SNP). They are easily recognizable after the amplification of the specific part of DNA, which includes the mutation and the incubation with the particular restriction enzymes. Furthermore, this procedure has not been used regularly, because of disadvantages such as requirements of a large DNA sample, time-consuming process and inability to identify all types of mutations (Barkaoui et al., 2007[2]).

#### Denaturing high performance liquid chromatography (DHPLC)

In 2000, two different papers were reported that genomic sequence variants and novel mutations of GSD Ia can be detected by denaturing high performance liquid chromatography (DHPLC) technique after PCR (Santer et al., 2000[48]; Lam et al., 2000[30]). DHPLC is a technique, which uses heteroduplex formation between wild-type and mutated DNA strands to detect mutations by reverse-phase liquid chromatography on a special column matrix. DHPLC is potentially a useful method for the mutation screening of a large number of samples. Santer et al. (2000[48]) have described the efficiency of DHPLC, for the detection and differentiation of exon 8 mutations (with almost 80% of patients carrying at least one exon 8 mutation) frequently encountered in a German GSD 1 *non*-A patients which is advantageous for a primary molecular genetic diagnostic approach. They concluded that the principal advantages of DHPLC are its semi-automated nature, with rapid results (a few minutes per sample), and the feasibility to collect eluted DNA for next analyses, but DHPLC alone cannot provide the details about the nature of mutations. Also, the sensitivity of the method is dependent on the temperature of analysis, the selection of which is dependent on operator experience (Santer et al., 2000[48]). Though nowadays analytical software programs have been developed for predicting the optimal temperature for DHPLC analysis, but it's still one of the challenging points of this technique (Santer et al., 2000[48]). Lam et al. (2000[30]) have reported the first prenatal diagnosis of GSD1b using DHPLC by screening fetal DNA for the G149E mutation. Their finding make a definite diagnosis of fetal GSD1b 14 min after PCR products were available for analysis. They concluded that DNA mutation analysis can be used in the prenatal diagnosis of GSD Ib and that DHPLC promises to be a robust technique for this prenatal molecular diagnosis (Lam et al., 2000[30]).

#### Allele-specific amplification combined with TaqMan fluorogenic probe (TaqMan-ASA)

To eliminate post-PCR steps and reduce errors, real-time PCR usage has increased in recent years. Allele-specific amplification (ASA) combined with a TaqMan fluorogenic probe (TaqMan-ASA) (Fujii et al., 2000[13]; Kojima et al., 2004[26]) was used to further confirm the known and novel mutation of GSD Ia. ASA is one of the most frequently practical diagnostic methods. It is based on the monitoring a mismatch between a template and a PCR-primer which reduces or prevents amplification. TaqMan-ASA monitors the efficiency of PCR amplification using allele-specific primers in real time (Zhu et al., 2012[64]). Two articles have used TaqMan-ASA for identification of GSD I patients (Fujii et al., 2000[13]; Kojima et al., 2004[26]). Fujii et al. (2000[13]) have devised TaqMan-ASA method for identification of prevalent point mutations of GSD Ia in Japanese patients. They screened a 727G>T mutation in *G6PC *gene because it is a major cause of GSD-Ia among Japanese patients. The high reproducibility and sensitivity of this technique indicates that the method may be safely applied to clinical diagnosis. Afterward, Kojima et al. (2004[26]) used a novel TaqMan-ASA method to facilitate the molecular diagnosis of GSD I in Japan, which detected the remarkably high prevalence of the two mutations in Japanese patients with GSD-I, 727G>T in type Ia and W118R in type Ib. Their results indicated that the combination of the two TaqMan-ASA methods, one for 727G>T and the other for W118R, could identify the majority of patients and facilitate the genetic testing of type Ia and Ib. Also, Zhu et al. (2012[64]) performed DNA sequencing and TaqMan gene expression assay for the coding region of the G6Pase gene in a Chinese patient with GSD Ia. They concluded that these techniques may be easily applied to detect point mutations in GSD I as a practical and clinical method after the identification of mutation spectrum in populations. The analysis fulfills all the requirements for diagnostic applicability, except the high cost of the instrument, which will perhaps decrease with prevalent application of the probe (Kojima et al., 2004[26]; Zhu et al., 2012[64]).

#### High Resolution Melting Analysis (HRMA)

Lately, high resolution melting analysis (HRMA), a simple real-time PCR-based method for detecting sequence variations for GSD Ia was developed. The basis of this method is that changes in amplicons of HRM are dependent on their DNA melting curves in the presence of saturating DNA binding dyes (Ezgu et al., 2014[12]). Therefore, changes in the melting curve of DNA duplexes for genotyping and variant scanning are investigated. Ezgu et al. (2014[12]) have recommended using the HRM analysis as a rapid molecular test for detection of known mutations in Fabery and GSD Ia diseases. They screened the most common mutation of G6PC gene, c.247C > T, in exon 2 that can cause GSD Ia among Turkish patients. They noted the difficulty in detecting homozygous mutants from the wild-type profile even with HRMA but homozygosity for mutation was clearly discriminated from the normal control samples. They concluded that compared with other presequencing mutation screening methods such as DHPLC, HRMA may be more sensitive and specific, even in homozygous mutant samples. The advantages of HRMA are a low cost technique, using samples directly for sequencing being a rapid technique compared with other mutation screening methods. However, some difficulty has been reported in detecting homozygous mutants from the wild-type profile (Ezgu et al., 2014[12]).

#### Deoxyribonucleic acid (DNA) microarray

In the last decade, the use of DNA microarray technique for diagnosis of genetic diseases has been reported (Xu et al., 2010[61]; Eminoglu et al., 2013[11]). The use of microelectronic-based techniques is increasing for diagnosis of genetic diseases day by day. DNA microarray has appeared as a choice for rapid genotyping large numbers of single nucleotide polymorphisms (SNPs), short tandem repeats (STRs), and small insertion and/or deletion mutations (Xu et al., 2010[61]). Microarray is a method based on scanning the data by laser. This method was established on hybridization of genomic DNA fragments with fluorescent-labeled probes and then sending the fragments to chip by electrical addressing. Finally, the results are obtained quantitatively (Eminoglu et al., 2013[11]). In the literature, two articles have reported the use of DNA microarray for detection of the most common mutations of GSD Ia patients (Xu et al., 2010[61]; Eminoglu et al., 2013[11]). Xu et al. (2010[61]) have developed the microarray technique as a rapid detection method for DNA-based diagnosis that is capable of identifying known mutations in the G6PC gene of Chinese patients. It is known that in some geographic regions and ethnic groups such as Turkish, Hispanic, Jewish, Japanese and Chinese, GSD Ia has allelic homogeneity. Based on this fact, Eminoglu et al. (2013[11]) have developed a new method by microarray technology which screened 12 most common mutations in the world, in 27 Turkish patients diagnosed for GSD Ia and the relation between detected mutations and clinical and laboratory findings. They suggested that microarray technology is totally cost-efficient and high-capacity compared to other methods and permits rapid analysis of known and prevalent mutations. It can successfully be used instead of screening the whole gene (Xu et al., 2010[61]; Eminoglu et al., 2013[11]). But, both papers concluded that microarray can only detect known mutations and may miss some novel mutations, however, DNA sequencing is required to specify the novel mutations in those patients negative results by DNA microarray (Xu et al., 2010[61]; Eminoglu et al., 2013[11]). 

## Conclusion

Despite of the increasing information about the gene structure of GSD I and the progress in molecular genetics, still there are no precise and cutting-edge recommended diagnostic tools. Among the molecular methods, DNA sequence analysis, especially NGS, provides accurate diagnosis and does not need tissue samples other than whole blood but the cost of the analysis has made researchers look for rapid and cheaper molecular screening techniques. Sensitivity and detection rate of NGS is nearly 100 %, so, NGS as the gold standard in combination with biochemical and clinical signs provides an accurate, high-throughput method of making genetic diagnoses of GSDs. With the reduction in sequencing costs, NGS is now the most accurate, cost and time efficient strategy for GSD I mutation analysis and diagnostic patients. With the development of third generation of sequencing (PACBIO and nanopure DNA sequencing), the cost, speed and accuracy of detection increase. 

## Disclosure statement

The authors declare that they have no conflicts of interest.

## Figures and Tables

**Table 1:**
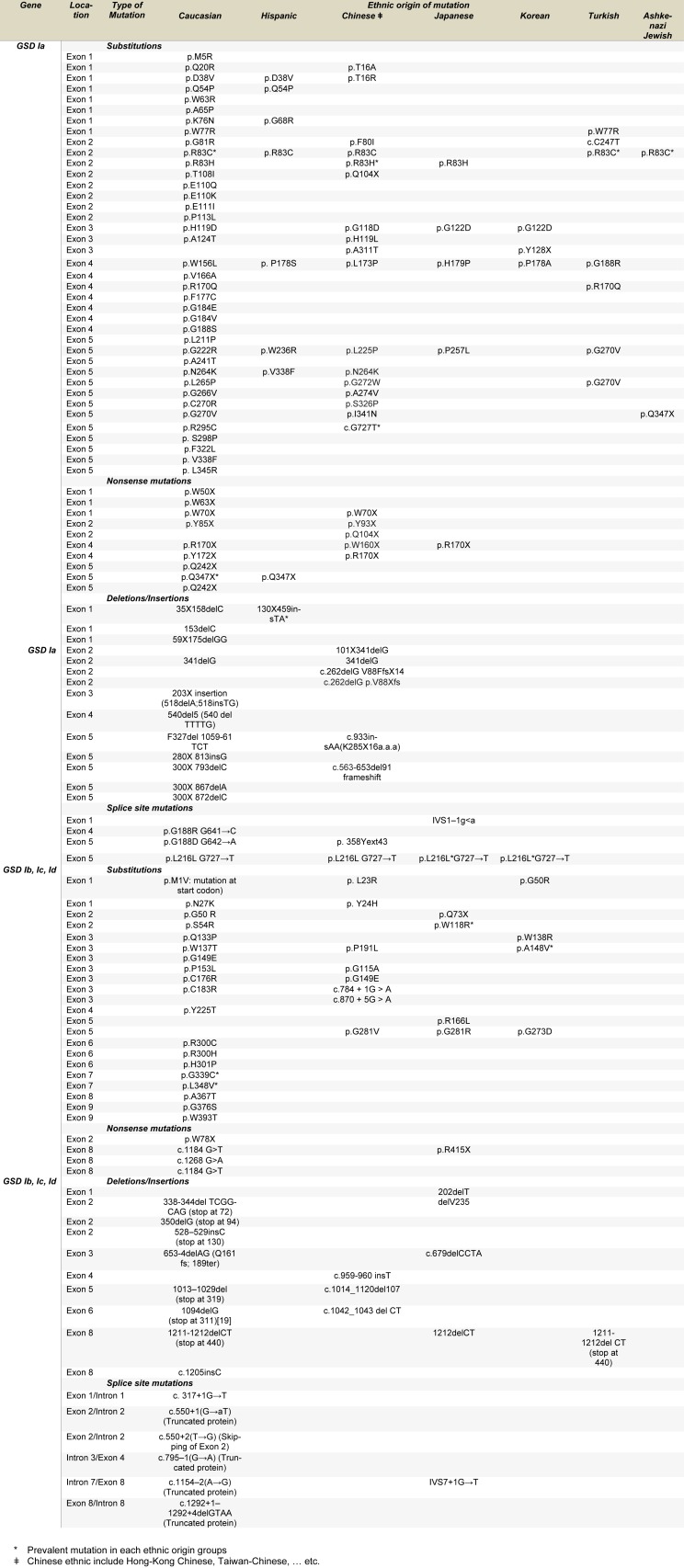
GSD I mutations identified up to date in different ethnic groups

**Table 2:**
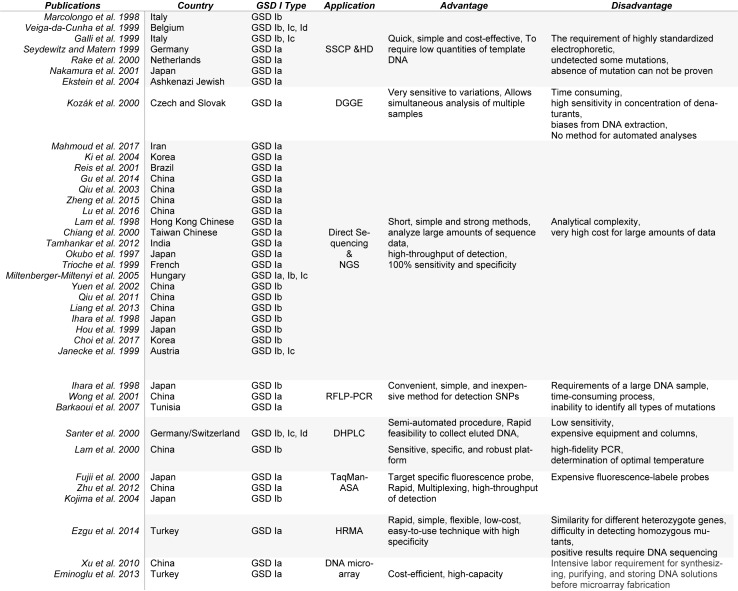
Summarized publications used in this work with advantages and disadvantages of molecular diagnostic tools
